# Optimization of diffuse Raman spectroscopy for in-vivo quantification of foreign body response in a small animal model

**DOI:** 10.1364/BOE.512118

**Published:** 2023-11-29

**Authors:** Max Dooley, Jeni Luckett, Morgan R. Alexander, Pavel Matousek, Hamid Dehghani, Amir M. Ghaemmaghami, Ioan Notingher

**Affiliations:** 1 School of Physics and Astronomy, University of Nottingham, University Park, Nottingham, NG7 2RD, UK; 2 School of Life Sciences, University of Nottingham, University Park, Nottingham NG7 2RD, UK; 3 School of Pharmacy, University of Nottingham, University Park, Nottingham NG7 2RD, UK; 4 STFC Rutherford Appleton Laboratory, Central Laser Facility, Research Complex at Harwell, UK Research and Innovation (UKRI), Harwell Oxford OX11 0QX, UK; 5 School of Computer Sciences, University of Birmingham, Birmingham, B15 2TT, UK

## Abstract

Diffuse Raman spectroscopy (DRS) allows subsurface molecular analysis of optically turbid samples. Numerical modeling of light propagation was used as a method for improving the design of an DRS instrument to maximize the signal to noise ratio (*SNR*) while ensuring safe laser exposure parameters required for *in-vivo* measurements. Experimental validation of the model was performed on both phantom samples and disks implanted postmortem to mimic the typical response to foreign bodies (formation of a fibrotic capsule around an implant). A reduction of laser exposure of over 1500-fold was achieved over previous studies whilst maintaining the same Raman collection rates and reaching the safe power density of 3 mW/mm^2^. The validation of this approach in a subcutaneous implant in a mouse cadaver showed a further improvement of 1.5-fold SNR, with a thickness limit of detection for the fibrotic layer of 23 µm, under the same acquisition times. In the animal body, a thickness limit of detection of 16 µm was achieved. These results demonstrate the feasibility of numerical model-based optimization for DRS, and that the technique can be improved sufficiently to be used for *in-vivo* measurement of collagenous capsule formation as a result of the foreign body response in murine models.

## 1. Introduction

Biomaterials play a key role in healthcare. Regardless of their endpoint application, implanted biomaterials trigger host immune responses, also known as foreign body responses (FBR). This response can lead to chronic inflammation, tissue damage, and fibrosis, resulting in device rejection and failure [1]. The standard method for evaluating FBR in animal models is histological assessment of *ex-vivo* tissue surrounding the implant. The thickness of fibrotic capsule and infiltration of immune cells (inflammation) are two key histological parameters [2,3]. The main drawback of histology is that it is an endpoint technique that requires excision of tissue. Obtaining time-course FBR data with sufficient statistical significance requires sacrificing a large number of animals, especially when considering the large spectrum of parameters typically required in the development of biomaterials and implants [4]. The collagen growth and formation of fibrotic tissue is of particular interest as it could give an early indication of device rejection once the initial inflammation has reduced.

While several imaging modalities can be used for *in-vivo* imaging in small and large animals (e.g. X-ray computed tomography [5], magnetic resonance imaging [6], ultrasound [7], optical coherence tomography [8], photoacoustic imaging [9]), they have limited molecular specificity and sensitivity for monitoring biomolecular changes in tissue related to FBR. Raman spectroscopy has been widely used for *in-vivo* monitoring of skin, including animals and humans [10]. In *ex-vivo* experiments, Raman spectroscopy can detect and image infiltration of immune cells during inflammation of dermis, and changes in collagen structure [11]. Raman spectra of inflamed dermis showed increased signals assigned to DNA bands, associated with the large number of immune cells (larger nuclei relative to their cytoplasm), and reduced collagen signal. In conventional Raman spectroscopy, when information is recorded predominantly from the surface of the sample, all photons reaching the detector travel in straight-line paths (ballistic photons), providing maximum spatial resolution. However, when attempting to detect molecular changes deeper under the surface of a sample with high level of optical scattering, such as tissue, both the spatial resolution and spectral contrast rapidly degrade with increasing depth. This occurs because the scattered photons will consist of both ballistic and diffuse photons (photons that underwent multiple scattering events). Therefore, *in-vivo* confocal Raman microscopy is typically limited to the outmost 100 µm layer of skin [12] and is not suitable for probing deeper into tissue.

The diffuse nature of photon migration in connective tissue for near-infrared light (785-1000 nm wavelength) makes diffuse Raman spectroscopy (DRS) ideally suited for probing deeper in and under skin, to measure the biomolecular processes during FBR. DRS can probe millimetres-centimetres deep into tissue [13,14]. DRS approaches have been demonstrated for monitoring and quantifying collagen in bone tissue [15] and cartilage grafts grown *in-vitro* [16]. A DRS technique based on spatial light modulators provided flexibility to optimise the configuration of the laser excitation and Raman detection points on the sample surface [17]. This allowed quantification of collagen density in samples implanted under the skin of rat cadavers, mimicking collagen deposition during healing of bone defects [17]. Although biologically relevant levels for sensitivity were obtained, the instrument used a laser power density ∼4-fold higher than the maximum permissible exposure (MPE) for skin (3 mW/mm^2^) [18].

In this paper we optimized the DRS instrumentation to maximize sensitivity when using laser power density at the MPE to allow *in-vivo* monitoring of collagen deposition during FBR. Computer modelling was used to simulate photon migration in tissue and optimise the configuration of laser excitation and Raman photon detection to target the sampling in the tissue volume adjacent to the implant. The modelling results were used to build an optimised DRS instrument, which was then tested on phantom samples to check the predictions for limit of detection for collagen, as a marker for the fibrotic tissue in FBR. The feasibility of performing *in-vivo* experiments for monitoring FBR was then investigated using mouse cadavers.

## 2. Materials and methods

### 2.1 Phantom and animal samples

**Fig. 1.**
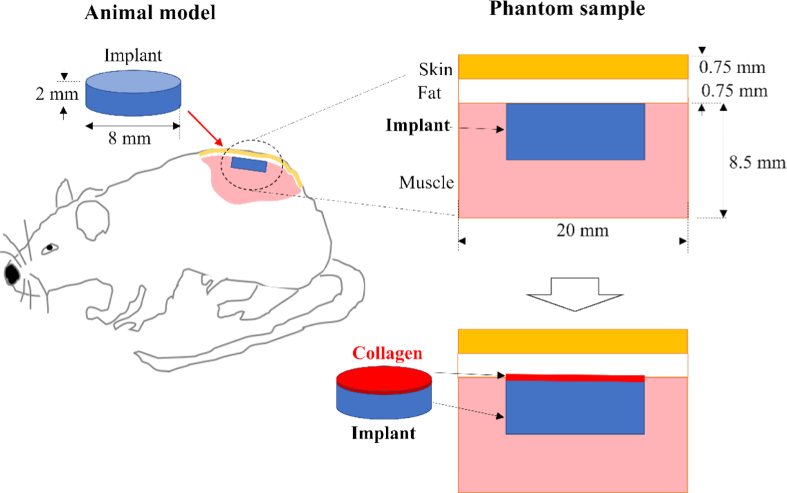
Schematic description of the mouse model for FBR (left) and the phantom sample (right). The implant was made of polystyrene discs coated with collagen layers (thickness 0-200 µm). The “Initial conditions” sample (right, top) contains the implant as it would be directly after being place subcutaneously mimicking the “day 0” condition of implantation. “FBR” sample (right, bottom) contains the implant coated with a collagen layer to mimic the growth of the fibrotic capsule.

Figure 1 shows the schematic description of the mouse FBR model and the phantom sample. The configuration of the phantom sample was selected to be biologically relevant, repeatable, and suitable for both computer modelling and experimental measurements. Polystyrene disks (8 mm diameter, 2 mm thickness) coated with a thin layer of collagen (thickness 0-200 µm) were initially placed on a thick muscle layer (chicken thigh) and covered with a layer of chicken skin. The rotation symmetry of the disk aided the search efficiency for computer modelling used for geometry optimization. The chicken skin (∼1.5 mm) consisted of a skin layer (∼0.75 mm) with a fatty layer (∼0.75 mm) behind. The overall sample was at least 20 × 20 × 20 mm^3^ in size to ensure the loss of light at the boundaries of the material were minimal. For the phantom samples each measurement was taken on a new sample to avoid the loss of collagen from the surface of the implant.

Collagen layers on the implants were created by depositing a solution of collagen on the top surface of the implant with a micro pipette. The collagen solution was 1 part collagen (bovine Achilles tendon powder, Sigma-Aldrich product code C9879-25 G) for ten parts 0.1 M potassium hydroxide. The collagen was left until fully dissolved before being pipetted onto the polystyrene disk. To make collagen layers that mimic different thicknesses of fibrotic capsule, different masses of the solution were added on the disks. It has been reported that the density of collagen around an implant is 400-500 µg/mm^3^ [19] while the concentration in normal skin is 150-250 µg/mm^3^ [20]. The mimicked thickness was calculated using the surface area of the implant and the density of collagen in a fibrotic capsule (500 µg/mm^3^). This value gave the thickness/mass ratio of deposited solution as 6 mm/g. Each implant was weighed before and after the solution was added then left to dry.

#### 2.1.1 Animal models

All animal experiments were approved following local ethical review at the University of Nottingham and performed under home office licence PP5768261. Female mice BALB/c 19-22 g were housed in individually vented cages under a 12 h light cycle, food, and water *ad libitum*.

After schedule 1 sacrificing, to insert the polystyrene disks subcutaneously a 10 mm long incision was made through the skin layer on the hip of the animal. Using a pair of tweezers, a 10 mm x 10 mm area of skin was lifted away from the muscle below it, creating a subcutaneous space 1.5 mm below the surface of the skin that the polystyrene disc could be inserted into via the incision. The skin was then moved back to its original position for the measurements.

### 2.2 Modelling and instrument optimization

The computational models were based on NIRFaster software, which was developed for modelling diffuse optical imaging and tomography [21]. NIRFaster utilises a finite element method (FEM) that models photon propagation, the forward problem, using the Diffusion Approximation of light propagation in tissue and then recovers the spatial distribution of the optical properties, the inverse problem and has previously been used for diffuse Raman tomography of *ex-vivo* canine bone [22].

The metric chosen for optimization was the signal to noise ratio (*SNR*) for the Raman signal from the collagen layer. The Raman signal for the collagen layer was calculated as the number of Raman photons in the 910–950 cm^-1^ band of the measured spectrum (after subtraction of a local linear baseline). The noise, which was dominated by photon shot noise, was calculated as the square-root of the total number of photons collected in this wavenumber region.

To calculate *SNR* with NIRFaster, two values where required: the intensity value generated by the model at the detector, and Raman spectra of all materials that made up the phantom samples. The material (Material) specific “signal intensity” from the model of a given sample (sample) was defined as $n_{sample}^{Material}$ . To obtain the values of the Raman intensities, reference Raman spectra were collected for all five materials that made up the phantom sample. The photon counts for the Raman signal were calculated using a reference spectrum as the number of photons in the 910–950 cm^-1^ range after subtraction of a local linear baseline, $I_{Raman}^{Material}$ . When total photon count was used to calculate the noise, the total number of photons in the 910–950 cm^-1^ range was calculated without any baseline subtraction for each material, $I_{Raw}^{Material}$ .

The Reference Raman spectra were measured with the free space Raman instrument, section 2.3.1. To match these reference spectra to the computer model, the measurements were taken with 900 s total integration time and 45 mW laser power at an excitation wavelength of 785 nm, the same parameters were used in the diffuse measurements. Reference spectra were taken in the backscatter Raman configuration with the excitation and detection point in the same position (0 mm offset).

For the experimental data, the signal from the collagen layer was calculated by subtracting the Raman spectrum of the “Initial Condition” sample from the Raman spectrum of the “FBR” sample, and calculating the area under the 910–950 cm^-1^ band after the subtraction of a local linear baseline. The noise corresponding to the collagen signal was dominated by the shot noise of the Raman spectra in the 910–950 cm^-1^ region. Because the signal from the collagen layer was calculated from the subtraction of two spectra, the noise from both spectra become additive, so the noise will be dominated by the value of the square root of the sum of the total number of photons from the spectra from the “FBR” sample and the “Initial Condition” sample in the 910–950 cm^-1^ region.

In the FEM model the signal of the collagen was calculated directly as $n_{FBR}^{Collagen}\cdot I_{Raman}^{Collagen}$ using the “FBR” sample. The noise was estimated as the square-root of the total photon count. This was calculated as to total photon count from all of the materials in both the FBR” sample and the “Initial Condition” sample. Thus, the SNR was calculated using the formula:

(1) $$SNR=\;\frac{n_{FBR}^{Collagen}\cdot I_{Raman}^{Collagen}}{\sqrt{\sum_{FBR}n_{FBR}^{Material}\cdot I_{Raw}^{Material}+\sum_{Initial\;Conditions}n_{Initial\;Conditions}^{Material}\cdot I_{Raw}^{Material}}}$$

#### 2.2.1 Calculating the optimal *SNR*

To reduce the search space for finding the optimal excitation/detection geometry, constraints were placed on the possibilities for laser excitation and Raman detection configurations to maximise *SNR*. First, the power density of the excitation laser was limited to the maximum permissible exposure for skin (3 mW/mm^2^). Because laser delivery to multiple points is a technically difficult task from instrument design point of view, both the excitation and detection areas were limited to simple geometric shapes, namely circles, disks, lines, and rectangles. Detection and collection areas also needed to be separated to obtain practical configurations for designing the Raman probe. The whole search space was limited to a 10 × 10 mm^2^ area on the sample surface, centred over the implant.

The FEM model was set up with a uniform mesh across the 3-D sample with 0.6 mm distance between nodes. In the area around the collagen layer, the distance between nodes was reduced to 0.08 mm to increase the resolution of the results. The simulation included a total of 441 excitation and detection points, respectively, uniformly spread on a 21 × 21 grid, from -5 mm to +5 mm in the *x* and *y* directions on the surface of the sample (*z *= 0). This created a 2-D surface for use to optimise the measurement geometry.

#### 2.2.2 Estimating the limit of detection from the *SNR* calculations

The “FBR” sample used in the FEM model used a thickness t=100μm for the collagen layer, generating a value for the signal to noise ratio denoted as $SNR_{100}$ . To calculate the thickness of collagen that is at the limit of detection, $t_{LOD}$ , we calculated the point at which the signal equals the noise amplitude. This will occur at the point that the collagen signal equal 1.645 times the noise [23] and therefore $SNR_{LOD}=1.645.$ Considering the signal from the collagen layer is directly proportional to its thickness, $t_{LOD}$ was be calculated using:

(2) $$t_{LOD}=\;\frac{1.645\;\cdot 100\mu m}{SNR_{100}}$$

### 2.3 Raman instrumentation and measurements

#### 2.3.1 Point-point diffuse Raman spectroscopy

A Raman instrument consisting of a single laser excitation point (0.1 mm diameter) and a single detection point (area 0.1 × 0.1 mm^2^, total signal of 11 channels of the spectrometer CCD with a slit width of 0.1 mm) was used for baseline measurements and evaluation of computer modelling results. This instrument was based on a previous free-space design and used galvanometers mirrors to generate adjustable spatial offsets between the laser excitation point and the point of collection for the Raman photons [24]. The collection point was always on the optical axis of the detection system so that the efficiency of collection did not change as the excitation offset changed. The objective lens for this instrument had 75 mm focal length, with 2-inch diameter optics for the full length of the optical path. The spectrometer was a Princeton Instruments Acton785 with an Andor Idus416 CCD detector with a pixel size of 10.6 × 10.6 µm^2^ and a slit width of 100 µm. The laser had a wavelength of 785 nm and power at the sample surface was 45 mW, which is equivalent to a power density of 5700 mW/mm^2^. Using the limiting aperture of 1.75 mm for 400-1400 nm wavelengths (23), provides a value of 29 mW/mm^2^ for the MPE, which is ∼1.5-fold lower than the power density used in the experiments. For simplicity and consistency when comparing power densities across multiple geometries used in this study, we will consider the true excitation area. To obtain sufficient *SNR* for the Raman spectra, the measurements had a total of 900 second integration time (sum of 18 spectra, 50 second integration time per spectrum).

#### 2.3.2 Linear fiber-based diffuse Raman spectroscopy

This Raman instrument utilised a laser with a wavelength of 785 nm delivered through a Powell lens, which created a line-shaped laser spot of 10 × 1.5 mm^2^. To ensure the power density was equal to the skin MPE limit (3 mW/mm^2^), the total laser power was set to 45 mW. The collection geometry comprised of two collection fibre bundles, each with their own 785 nm long pass filter (Semrock LP02-785RE-25). The fibres bundles (Thorlabs BFA105LS02) each had seven 105 µm core diameter fibres set linearly in a 1 × 7 array. These were imaged onto the surface of the sample using a 1-inch diameter lens of 50 mm focal length, and a then projected onto the fibres with a lens with focal length 30 mm. This provided a total collection area of 0.20 mm^2^, which is approximately 20-fold higher the collection area of the Point-Point instrument. The spectrometer used was a Princeton Instruments ISO320 with a Pixis2048 CCD. A total of 160 channels were summed on the CCD to combine the spectra from al l4 fibres. Pixel size on the Pixis2048 was 7.4 µm (5.3 µm x 5.3 µm). The integration time was 900 seconds (9 individual measurements, each of 100 s). The total measurement time was 20 minutes which is compatible with current relevant procedures was imaging small animals under anaesthetic [25].

### 2.4 Data analysis

Before calculating the difference spectra between the samples mimicking FBR (contained polystyrene disk coated with collagen) and the sample mimicking the initial condition (polystyrene disk with no collagen layer), the individual measured spectra were normalised such that the polystyrene 1004 cm^-1^ band was between 0 and 1. The normalised spectra from the “initial condition” sample were subtracted from the normalised spectra corresponding to the “FBR” samples. For the calibration model, sixteen difference spectra were used to build a Partial-Least Squares (PLS) model and validated using the leave-one-out method to predict the thickness of the collagen. The PLS model was created using the “Statistics and Machine learning toolbox” from MATLAB 2022b. A PLS model is a linear model that the finds the dimensions in the spectral data that maximise the variation in the training data creating components that have spectral features that best correlate with the physical property, in this case Collagen thickness, that the model predicts. The dataset contained two samples with collagen thicknesses of 10 µm, 20 µm, 40 µm, 60 µm, 80 µm, 100 µm, 150 µm, 200 µm each. The PLS model was then applied to a second set of Raman spectra measured from a new batch of samples with the same collagen thicknesses. The root mean square error (RMSE) of the residuals of the prediction from the known values was calculated and used as a value for the limit of detection.

## 3. Results

### 3.1 Optimisation of laser excitation and Raman detection configurations

First computer modelling was used to determine the optimal configuration of laser excitation and Raman detection points to increase the sensitivity of the DRS measurements for collagen concentration.

**Fig. 2.**
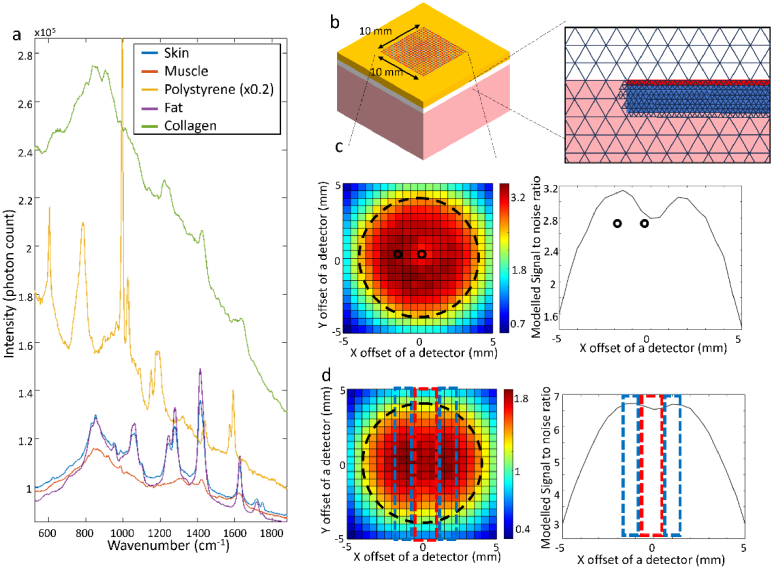
a) Reference Raman spectra of the materials making up the phantom sample. The intensity of the polystyrene spectrum was scaled down 5-fold. b) A 3-D representation of the simulated phantom sample showing the 21 × 21 array of excitation sources and detectors on the sample surface (red and blue dots), arranged in a 10 mm x 10 mm grid. The stylised illustrations of the FEM mesh around implant region (the black lines show the links between the mesh node points) c) *Left:* A surface plot showing the *SNR* for each individual detector corresponding to a single point laser excitation at the centre of the sample, position (0,0). The circle in dotted black line shows the position of the implant. Black solid line circles show the positions of the source and detector for maximum *SNR*. *Right*: The computed *SNR* as a function of the spatial offset (*x*) along the central *y* = 0 mm offset line with a central excitation point. d) *Left*: a surface plot showing the *SNR* recorded at each individual detector point when using a single line laser excitation (indicated by the red dotted line). *Right*: The SNR calculated when integrating the Raman signal for the detection points indicated by the two blue rectangular areas.

Figure 2(a) shows the reference Raman spectra for the materials that make up the phantom sample. The Raman spectrum of polystyrene shows the highest intensity, in particular the band at 1004 cm^-1^ corresponding to the ring breathing mode of the carbon ring [26]. There is also a band that overlaps with the 1450 cm^-1^ peak from the biological components of the sample corresponding to the C-H_2_ scissoring. The 1634 cm^-1^ band corresponds to the ring skeletal stretch is at lower wavenumber compared to the Amide I band in collagen. The Raman spectra of skin, fat, muscle and collagen show the key Raman bands typical to biological molecules. The Raman spectrum of collagen shows the characteristic bands 850 and 930 cm^-1^ (assigned to proline and hydroxyproline in collagen), the 1000-1150 cm^-1^ band (assigned to C-C and C-O stretching [27]), the 1200-1400 cm^-1^ band (assigned mainly to Amide III in proteins [27]), the 1450 cm^-1^ band (assigned to C-H deformation vibrations in proteins and lipids [27]) and the 1660-1690 cm^-1^ band (Amide I [28]). Therefore, the spectral region 800-980 cm^-1^ avoids the intense 1004 cm^-1^ ring breathing band in the Raman spectrum of polystyrene, as well as minimising overlap with the Raman bands of other biological molecules.

Figure 2(b) shows a 3D schematic of the phantom sample showing the positions of the laser excitation and Raman detectors for the computer model, including the FEM mesh. The simplest configuration investigated consisted of a single excitation laser spot at the centre of the sample (*x *= 0, *y *= 0). Figure 2(c) presents the computed *SNR* at each detection point on the surface of the sample, showing a maximum value of *SNR*_100 _= 3.2 at a radial offset of 1.5 mm around the (0,0) point excitation. From Eq. (2) we calculated the limit of detection as 1.645⋅100/3.2=51μm . This result predicts that a Point-Point instrument would provide a biologically relevant sensitivity for measuring changes in collagen thickness caused by FBR. However, a 45 mW laser power focused on a 0.1 mm diameter spot would lead to a laser power density greater than the MPE by a factor of 1.5 and greater than the illumination intensity limit for diffuse excitation by a factor of ∼2000 if the actual area of illumination area is considered.

Figure 2(d) shows that when the laser beam was expanded in a line (10 mm x 2 mm), two regions of high *SNR* were observed, either side of the *x *= 0 mm line. The modelling experiments were calibrated to use the same laser power as for the Point-Point instrument (presented in Fig. 2(c)), such that the results could be directly compared. The advantage of expanding the laser power in a line is that the same number of photons can be used to excite Raman scattering while reducing the power density to 3 mW/mm^2^, which is equal to the MPE for skin. The regions of high *SNR* were found directly above the implant disk in the *z* direction. When the collection of the Raman photons was integrated along a rectangular area (length 10 mm along the *y* axis, width 2 mm, including 21 collection points) parallel to the laser excitation line, the maximum value of the *SNR*_100_ was 6.7 and corresponded to a 1.5 mm offset from the *x = *0 mm axis. When using both rectangular areas for collection of Raman photons, the value of the total *SNR*_100_ was 9.5, Thus, the predicted limit of detection for collagen thickness when using this measurement configuration was calculated as 1.645⋅100/9.5=25μm .

### 3.2 Instrument development and calculation of limit of detection for collagen thickness using phantom samples

**Fig. 3.**
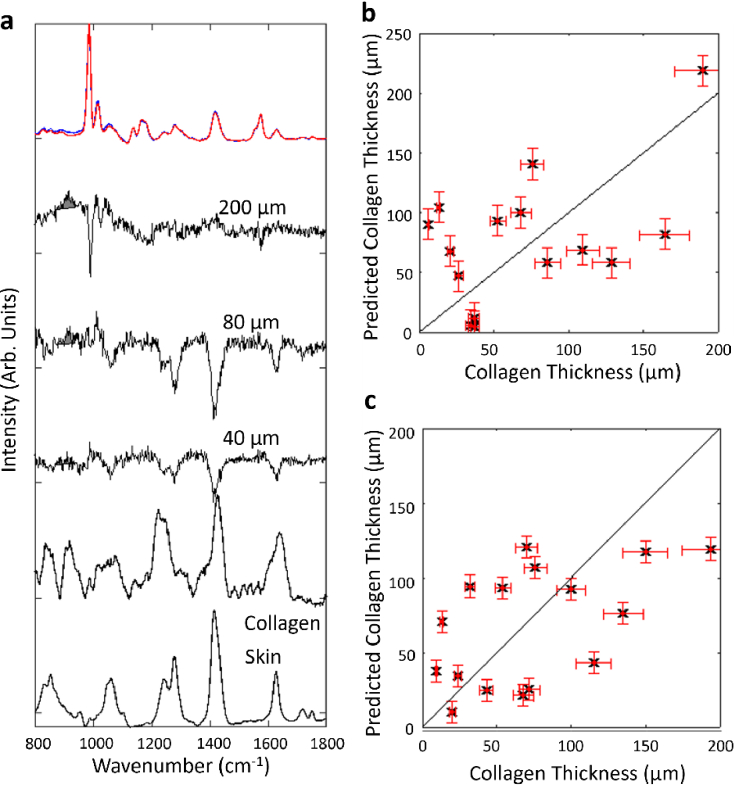
a) Raman spectra measured for the phantom sample (top) representing the Initial conditions (no collagen) sample (Red) and the sample containing 200 µm collagen (Blue). The black spectra show the difference spectra for phantom samples containing 40, 80 and 200 µm thick collagen layers. The shaded grey area indicates the 930 cm^-1^ band assigned to collagen. Raman spectra of collagen and skin are included for reference, Spectra are shifted vertically for clarity. b) Prediction results for the collagen thickness from the training set showing a leave-one-out validation. Vertical error bars show the RMSE of the PLS model on the training data set and horizontal error bars indicate the uncertainty on the manufactured collagen thickness (10%). The black line shows the prediction line when the prediction value is equal to the actual thickness. c) Results of the independent testing of the PLS model for collagen thickness. The black line indicates a perfect model when the prediction values are equal to the actual thicknesses.

Baseline experiments on phantom samples were carried out first using the Point-Point instrument (single laser point for excitation and a single spot for detection of the Raman photons) (Fig. 3(a)). The laser power was set to 45 mW, equal to the value used in the computer models.

Figure 3(a) shows examples of the measured spectra and computed difference spectra from three samples with collagen thicknesses of 40 µm, 80 µm and 200 µm respectively. The blue spectra are the measurements taken with initial conditions (no collagen layer), and the red spectra show the measurement taken with the polystyrene spectra coated with collagen, mimicking FBR. The calculated difference spectra show a correlation between the intensity of the 930 cm^-1^ band and the thickness of the collagen layer. Figure 3(b) shows the results of the PLS model leave-one-out cross-validation on 16 samples used for training the model (range of collagen thicknesses 10 - 200 µm). The prediction RMSE of the collagen thickness for the individual samples in the training dataset was 41 µm. The PLS model was tested on an independent set of samples (Fig. 3(c)). The results of the independent test showed a RMSE of 44 µm, in close agreement with the cross-validation results.

The results of this validation test had a more improved limit of detection (44 µm) than predicted from the FEM modelling (51 µm). PLS is a multivariate approach that used the full spectra to create the prediction model. The calculations of *SNR* and t_LOD_ focussed on the 910–950 cm^-1^ band as this was the band that had the clearest correlation with collagen concentration when inspecting the reference spectra. Using the total spectra increases the total photon count which can improve both signal and noise. The multivariate approach of PLS balanced the two of these to maximise the signal to noise ratio improving the limit of detection.

**Fig. 4.**
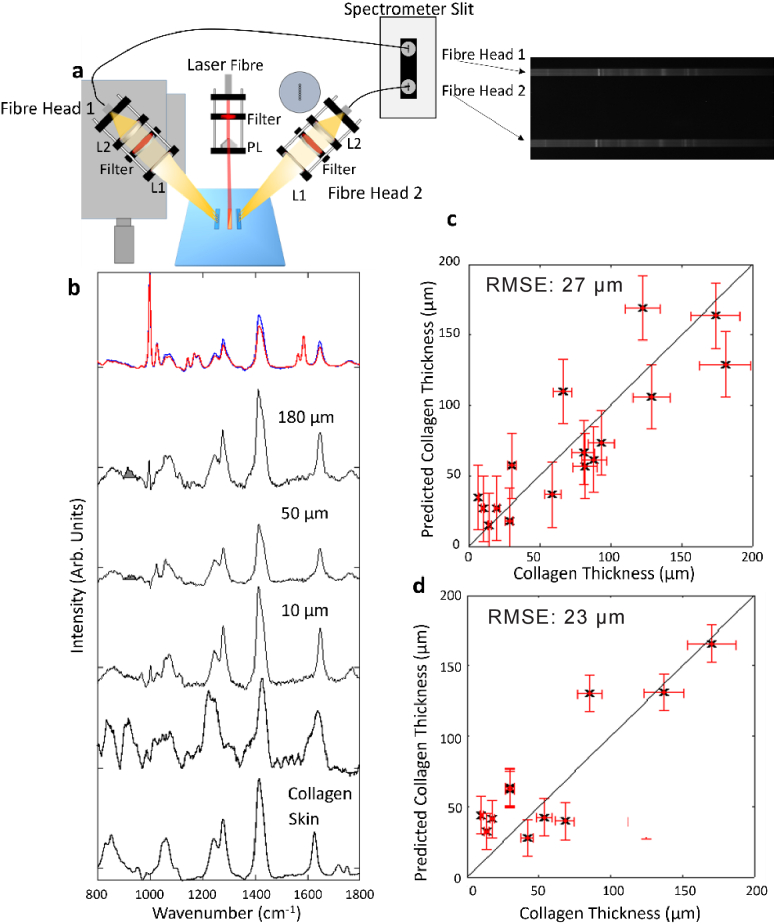
a) a) A schematic of the linear DRS instrument. The central red shows the optical fibre delivering the laser beam to a Powell lens to create a line-shaped laser beam. on the sample surface. The two fibre heads are shown symmetrical on translation stage to allow them to be focused on the two linear detection areas parallel to the laser line. The yellow area shows the paths of the collected Raman light focused on the two linear fibre bundles. The Blue surface shows the sample surface on which the enclosed blue areas show the collection areas, and the red areas shows the excitation area. The labelled Spectrometer slit shows the configuration of the two fibre bundles arranged vertically on the spectrometer slit and CCD b) Raman spectra measured using the optimised Linear DRS Raman instrument for the phantom sample representing the Initial conditions (no collagen) sample (Red) and the sample containing 200 µm collagen (Blue) shown normalised as they are used withing the PLS training model. The black spectra show the subtracted spectra for phantom samples containing 10, 50 and 180 µm collagen. The shaded grey area shows the 930 cm^-1^ band assigned to collagen. Raman spectra of collagen and skin. All spectra are shifted vertically for clarity c) PLS prediction results for the collagen thickness from the training set showing a leave-one-out validation. Vertical error bars show the RMSE of the PLS model on the training data set and horizontal error bars indicate the uncertainty on the manufactured collagen thickness (10%). d) Results of the independent testing of the PLS model for collagen thickness.

The achievement of improved *t*_LOD_ set the groundwork for the development of a new instrument based on the results from the computer modelling Fig. 2(d). The design of the linear instrument is presented in Fig. 4(a). The collection area of 0.202 mm^2^ was equivalent to twenty of the 0.1 mm x 0.1 mm detention areas in the Point-Point instrument. The detection points were spaced evenly from – 0.5 mm to 0.5 mm due to the spacing of the individual fibres at the head of a fibre bundle. Using the modelling results for a linear excitation area (Fig. 2(d)), the *SNR* for seven detection points was calculated by summing the signal from the individual detection points included in the area covered by the seven fibres. This calculation provided an *SNR*_100 _= 4.0, predicting a limit of detection of 41 µm.

The Raman measurements recorded with the Linear fibre-based DRS instrument using a laser power density of 3 mW/mm^2^ (equal to the MPE for skin) are presented in Fig. 4. Figure 4(b) shows example of normalised Raman spectra with and without collagen, and calculated difference spectra from samples with collagen thicknesses of 10 µm, 50 µm and 180 µm, respectively. Compared to the Point-Point spectra in Fig. 3(a), the *SNR* in the difference spectra is significantly higher (in particular when inspecting the spectral regions with no visible Raman bands, such as the region around 1500 cm^-1^). Figure 3(c) presents the leave-one-out cross-validation results for the training data set using the PLS model. The RMSE for this PLS model was 27 µm. This result represents a significant improvement over the Point-Point instrument Fig. 4(d) presents the results of the independent testing of the PLS model, confirming an RMSE of 23 µm.

This improvement was greater than the improvement predicted from the modelling ( $t_{LOD}=41\mu m$ ). A number of changes in the instrument design (improved detector sensitivity), such as the move from free space to fibre bundle collection would account for some of this discrepancy. It is also possible that as the *SNR* improved, differences between the parts of the spectra of collagen and skin that overlap became distinguishable. For instance, while both collagen and skin have a strong CH_2_ scissoring peak at 1450 cm^-1^, there are differences in the shape of these band between the two materials. As *SNR* improves a greater number of these subtle spectral changes can be incorporated into the prediction model, compounding the improvement in *SNR* and *t*_LOD_.

In both Fig. 3(a) and Fig. 4(b) the computed difference spectra contain very few spectral features corresponding to the polystyrene disks. This indicates that the normalisation method using the 1004 cm^-1^ band is effective in eliminating the Raman bands across the whole spectral region, and that the method could be applied to other implant materials.

### 3.3 Proof-of-concept measurements on mouse cadavers

**Fig. 5.**
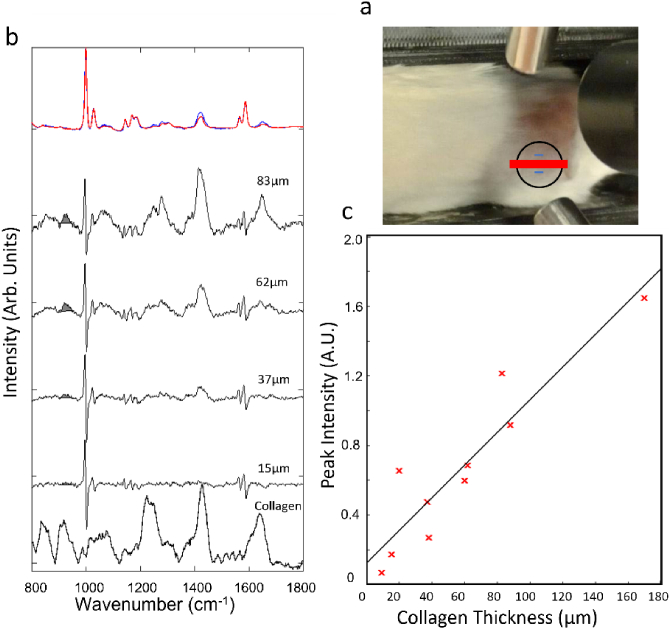
a) Photograph showing the orientation of the mouse on the optimised linear DRS instrument. Highlighted with a black line and red line are the positions of the implant with the laser excitation line. The short blue lines show the Raman collection areas. b) Typical normalised spectra corresponding to the initial conditions sample (Red) and sample with collagen (Blue). The black spectra under each shows the subtraction of the initial condition from the FBR spectra from the four thicknesses added to the right hip of mouse one. The shaded grey area shows the 930 cm^-1^ band assigned to collagen. Collagen reference spectrum is included. Spectra are shifted vertically for clarity. c) The area under the 930 cm^-1^ peak for each subtracted spectra plotted against the thickness of the collagen layer in samples.

Based on the results obtained with the optimised linear fibre-based DRS instrument, proof-of-concept experiments were carried out using mouse cadavers. These experiments represent a required step prior to progressing to full-scale *in-vivo* trials. The aim of these experiments was to test the applicability of the results and *t*_LOD_ predictions obtained using the phantom samples. For these experiments, polystyrene disks coated with collagen layers were placed in a pocket under the skin of mouse cadavers, in the area on the hip (Fig. 5(a)). Similar to the measurements using phantom samples, a disk with no collagen was used to mimic the initial conditions. Disks with increasing collagen thickness were added in stages to mimic the increased growth of the fibrotic capsule over time. Figure 5(a) shows typical normalised Raman spectra and computed difference spectra corresponding to four thicknesses of collagen: 15 µm, 37 µm, 62 µm, and 83 µm. Significant differences were observed between the Raman spectra obtained from the mouse cadavers and the phantom samples using chicken skin, in particular in the 1200-1400 cm^-1^ region. The Raman bands were approximately half the size relative to the polystyrene normalisation in the spectra from the mouse cadavers compared to the spectra from the chicken phantoms. Commercially available chicken are bred for high fat content explaining the large contribution in this spectral region.

The 1200-1400 cm^-1^ region region is dominated by the amide III vibrational modes (NH_2_ and C-N) in proteins and a combination of = C-H bend (1250-1280 cm^-1^) and CH_2_ twisting (1295- 1305 cm^-1^) in lipids. The spectra from the phantom sample show overall higher intensities for all these bands, as well as a relative higher contribution of the CH_2_ twisting (1295- 1305 cm^-1^) compared to the Amide III bands of proteins. The findings are consistent with the facts that the mouse skin was significantly thinner and had a lower concentration of lipids compared to the chicken skin. Because of these differences, the PLS model trained on the chicken skin phantom samples was not suitable to predict the collagen thickness for the mouse cadavers. Nevertheless, the computed difference spectra in Fig. 5(b) show that increasing the thickness of collagen led to an increase in intensity of the 850 cm^-1^ and 930 cm^-1^ Raman bands assigned to collagen. Figure 5(c). shows a strong correlation between the area of the 930 cm^-1^ Raman band and the thickness of collagen layer, with an R^2 ^= 0.86. The RMSE of the area under the peak against the collagen thickness was 0.0195 Arb. Units./µm. Using the equation of the line of best fit in Fig. 5(c) this corresponds to $t_{LOD}$ = 16 µm. This indicates that the difference in physical and chemical properties of a mouse model of FBR compared to the chicken phantom samples may be highly conducive to greater *SNR* that was achieved in the phantom measurement in Fig. 3 and Fig. 4.

## 4. Discussion

This paper reports the use of computer modelling to optimise diffuse Raman measurements for monitoring the collagen deposition that are part of the FBR triggered by subcutaneous implantation of biomaterials in a murine model. Computer modelling of light propagation in tissue was used to maximize the sensitivity and spectral contrast by increasing the optical throughput and optimizing the spatial configuration of laser excitation and detection points on the sample surface. The modelling results indicated an optical design based on line-shaped laser excitation and symmetric line-shaped detection, predicting a limit of detection ( $t_{LOD}$ ) for collagen thickness of 25 µm, when using a laser power density equivalent to the maximum permissible exposure (MPE) for skin (ANSI Z136.1-2007: 3 mW/mm^2^ for continuous illumination at 785 nm wavelength). After developing a linear DRS instrument based on this optimal design, experimental data indicated an $t_{LOD}$ =23 µm, confirming the predictions from modelling.

The agreement between modelling and experimental data is an important finding. In previous work, modelling was used to obtain a 2.2-fold improvement in spectral contrast for spatially offset Raman spectroscopy measurements of layered polymers samples over backscatter Raman spectroscopy [24]. In the context of FBR, modelling allowed significant improvements in the limit of detection for collagen thickness compared to values reported in the literature [19]. Previous work indicated a limit of detection 0.160 g/cm^3^ of collagen measured through a layer of skin ∼1.5 mm thick. Considering the diameter of the defect (6 mm), this corresponded to a total collagen mass of 0.009 g, which would be equivalent to a 90 µm thick layer [19]. Nevertheless, the results were achieved with a power density ∼1500-fold higher than the illumination intensity used in the linear DRS instrument developed in this paper.

The resulting sensitivity increase was also confirmed on mouse cadavers, after inserting polystyrene disks coated with collagen layers of 10-200 µm. The results indicated an *t*_LOD_ = 16 µm, improvement likely associated to the fact that mouse skin was thinner than the skin layer used for the phantom sample.

The limit of detection demonstrated in this study comes close to the levels required for monitoring FBR in small animal models. Recent studies investigating FBR at 28 days after implanting subcutaneously sections of medical-grade silicone urinary catheter tube (2.7 × 5 mm^2^, cut longitudinally in half), reported 40 ± 30 µm thick collagen layer surrounding the implant [3]. The results presented in this study indicate that the Linear DRS instrument has the sensitivity to detect the formation of the fibrotic capsule caused by FBR in such an animal model. Further improvements in sensitivity could be achieved by increasing the optical throughput of the instrument, in particular by increasing the number of the collection fibre optics. The development of custom fibre optics probes to cover the entire 10 mm x 1 mm collection area indicated by the computer model would increase the optical throughput by a factor of 50x, improving further the *SNR*. Such further improvements would increase the sensitivity of the DRS measurement, potentially allowing detection of biomolecular changes occurring at the very early stages of FBR.

## 5. Conclusion

This study reports the feasibility of computer modelling for optimizing diffuse Raman spectroscopy for *in-vivo* measurements and achieved relevant levels of detection while using laser power densities within the maximum permissible exposure for skin. The limits of detection for collagen thickness predicted by the computer modelling were confirmed experimentally using phantom samples and mouse cadavers. Overall, the results indicate the feasibility of conducting *in-vivo* time-course measurements to monitor FBR in small animals. The ability to monitor FBR non-invasively, on the same animal, would provide high quality longitudinal data with ethical and economic benefits by reducing the number of animals in research.

## Data Availability

Data underlying the results presented in this paper are not publicly available at this time but may be obtained from the authors upon reasonable request.
